# Physiological Effects of l-Theanine on *Drosophila melanogaster*

**DOI:** 10.3390/molecules181113175

**Published:** 2013-10-24

**Authors:** Hui Yang, Wenzhe Li, Huiyi Yu, Ruiqi Yuan, Yang Yang, Kingston Pung, Ping Li, Lei Xue

**Affiliations:** Department of Interventional Radiology, Shanghai 10th People’s Hospital, Shanghai Key Laboratory of Signaling and Disease Research, School of Life Science and Technology, Tongji University, Shanghai 200092, China ; E-Mails: yangh.g348@gmail.com (H.Y.); lwz@tongji.edu.cn (W.L.); cloverlife@163.com (H.Y.); rickyagent47@sina.com (R.Y.); yyang1206@tongji.edu.cn (Y.Y.); kpung12@gmail.com (K.P.)

**Keywords:** l-theanine, *Drosophila melanogaster*, locomotion, courtship, starvation

## Abstract

Green tea has been consumed as the most popular drink in East Asia for centuries, and is believed to have a wide range of health benefits. l-Theanine, the major component of the free amino acids in green tea, has been reported to display neuronal protection and tumor inhibition *in vitro*, but its physiological effects on animal development and behavior remain elusive. In this report, we used *Drosophila melanogaster*, the fruit fly, as a model organism to investigate the physiological effects of l-theanine. Flies were fed with three different concentrations of theanine as a dietary supplement after eclosion, and were examined for a variety of physiological parameters at different time points. We found theanine treatment results in significantly increased locomotion and courtship ability, and decreased resistance against wet and dry starvation in males, but not in females. Furthermore, theanine application diminished UV tolerance in females, but not in males. However, we did not perceive distinguishable effect of theanine on animal development, life span, weight, and tolerance of heat and anoxia. This work represents the first comprehensive physiological investigation of l-theanine at the whole animal level, and shall shed light on the mechanistic study of theanine in the future.

## 1. Introduction

**Figure 1:**
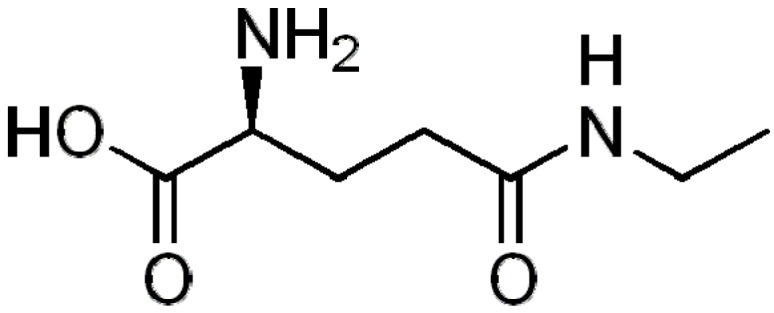
Chemical structure of l-theanine.

Green tea, originated from China, has been one of the most popular beverages in East Asia for centuries. It was also regarded by ancient Chinese as a kind of panacea for many different diseases. Scientific experiments and medical studies over the past few decades have revealed that green tea contains abundant nutrients with multiple health benefits [1]. Theanine, first isolated in the 1940s, accounts for 40% to 60% of the free amino acids in green tea [2]. Pure theanine presents as white needle-like crystals that are very soluble in water. As an acetanilide-group compound, it maintains the l-configuration under natural conditions (Figure 1) [2].

Theanine is a taste component of tea, producing a caramel flavor and aroma that helps to alleviate the bitterness of caffeine and the astringency of tea polyphenols [3]. Toxicology and safety evaluation tests suggest that theanine is a safe, non-toxic food additive. Hence, theanine was certified as a Generally Recognized As Safe (GRAS) material by the U.S. Food and Drug Administration (FDA) in 1985, and has been widely applied as a flavor modifier in the food industry. The synthesis and extraction of l-theanine has experienced large-scale technological advancement since 1950, with multiple methods now available including chemical synthesis, microbial fermentation and tea callus culture [4,5], thus expend the applicative potential of l-theanine as a drug and food addictive.

After oral administration, l-theanine is easily absorbed into the bloodstream and transported to major organs, including the brain [6]. Reports revealed that l-theanine is an effective antihypertensive drug that significantly reduced the systolic, diastolic and mean blood pressure in spontaneously hypertensive rats without any influence on normal rats [7,8]. Consistently, theanine was found to reduce blood pressure and to antagonize the effect of caffeine-induced blood pressure increases in humans [9].

Theanine was also reported to refresh the mental state through the generation of alpha brain waves and the regulation of dopamine and serotonin levels in the brain [10]. In addition, it was shown that l-theanine could increase the density of certain brain neurotransmitters, promote the formation of nerve growth factors, and accelerate the development of the central nervous system [11]. Consumption of theanine is closely associated with the reduction of anxiety and improvements of learning and memory ability in humans and rats [12,13,14,15]. Moreover, as a natural antioxidant, theanine exerts a wide range of neuro-protective effects against chronic restraint stress-induced cognitive impairments in mice [16], amyloid precursor protein-triggered neurotoxicity *in vitro* [17,18], amyloid beta-induced neuronal cell death and memory impairment in mice [19], glutamate receptor agonist-mediated brain injury in the rat model of stroke [20], and focal cerebral ischemia in mice [21].

As a derivative of glutamine, theanine disturbs its metabolism as a competitor, and thus inhibits the growth of several tumor cell lines, including human non-small cell lung cancer cells and leukemia cells [22,23]. In addition, theanine was shown to induce apoptosis in breast, colon, hepatoma and prostate cancer cell lines, and to inhibit tumor cell migration [24,25,26,27]. Administration of theanine could also enhance the immune response by increasing the glutathione level [28,29]. As an antioxidant, theanine was reported to extend lifespan and promote paraquat resistance in *C. elegans* [30], and reduce body weight and liver triglyceride levels in mice and humans [31,32].

With all of these medical potentials and application advantages, it is highly plausible that l-theanine might provide an opportunity to deal with many human diseases, yet its physiological function and molecular mechanism remains poorly understood. *Drosophila* has been used as an excellent animal model to investigate the molecular mechanism and potential treatment of many human diseases. However, to our knowledge, there are no detailed reports on the physiological effects of l-theanine on *Drosophila*. In this work, we included three different concentrations of l-theanine as a dietary supplement into flies’ food, and investigated the effects of l-theanine on fly development, lifespan, weight, locomotion, courtship and their tolerance to wet and dry starvation, UV, heat, and anoxia challenges.

## 2. Results and Discussion

### 2.1. Theanine Enhances the Locomotion of Drosophila Males

**Figure 2:**
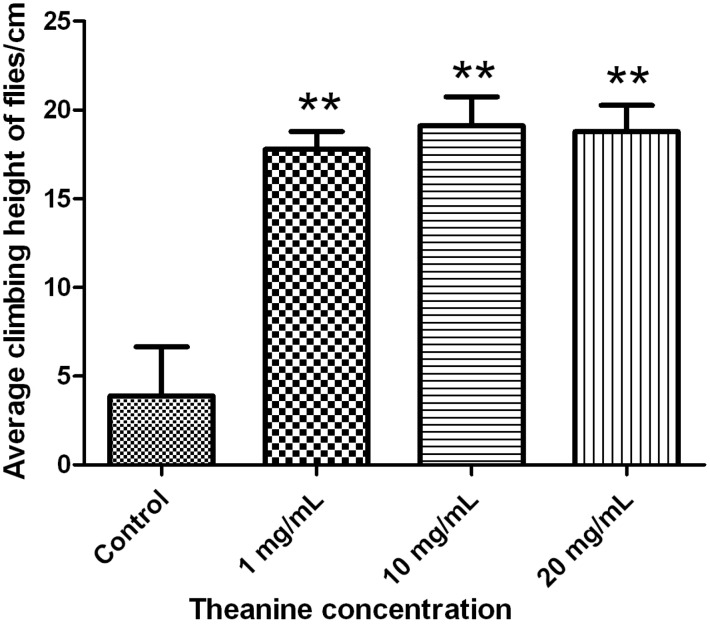
l-theanine treatment improves climbing ability of *Drosophila* males. 40-day-old males were placed in a climbing vial and their climbing heights were recorded in 20s. The data were calculated as mean ± standard deviation, ** *p* < 0.01* versus* control, t-test.

The wall climbing ability has been commonly employed to measure the locomotor behavior in *Drosophila*. We found that 40-day-old male flies of all three treatment groups displayed better climbing ability by reaching a height of 20 cm in 20 s (Figure 2). 

On the contrary, the average height that the control group reached was less than 5 cm, suggesting that theanine could enhance the locomotion of male flies. However, a similar effect was not observed in females (Figure S2A), implying a sex-dependent neurological consequence of theanine treatment. It has been reported that oral administration of cystine and theanine can improve locomotor activity and food intake in mouse, and enhance immune responses in mouse and humans [33,34]. It has also been suggested that athletes should take cysteine and theanine during high-intensity and high-frequency training to reduce intense exercise-associated symptoms [35,36]. Despite the synergistic anti-fatigue function reported for cystine and theanine, theanine alone failed to exhibit such effect in mammals. In the present study, however, we found theanine alone was able to enhance the locomotor activity in *Drosophila*. One explanation for the discrepancy is that the actual effect of theanine in *Drosophila* is not to increase the climbing ability, but rather to slow down the declining locomotor activity associated with aging. Nevertheless, such a possibility seems implausible given the fact that dietary supplements of theanine at the reported concentrations failed to provide any anti-aging benefit by extending the lifespan of *Drosophila* (Figure S1A and B). Further studies shall be required to investigate the underlying mechanism of the anti-fatigue performance of theanine with or without cysteine in different animal models.

### 2.2. Theanine Enhances the Courtship of Drosophila Males

**Figure 3:**
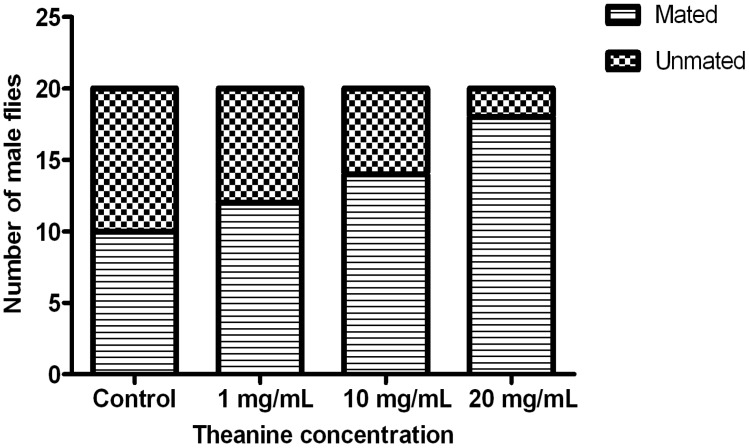
l-theanine treatment improves courting ability of *Drosophila* males. Proportion of mated males within two h in the group was steadily increased with theanine concentration. Statistical analysis was performed with chi-square trend test, *p* < 0.05.

Upon treatment of increased theanine concentration, 30 day-old males exhibited an improved courtship behavior that resulted in a stable enhancement in successful mating with virgin females within a certain period of time (Figure 3). This effect is consistent with theanine’s ability to enhance male locomotor behavior (Figure 2), which plays a dominate role in the courting process.

### 2.3. Theanine Reduces the Starvation Tolerance of Drosophila Males

**Figure 4:**
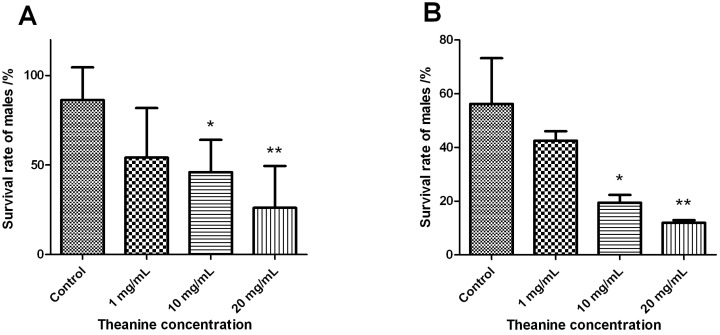
Reduced starvation tolerance of male flies fed with l-theanine. Survival rate of males after wet (A) and dry (B) starvation, * *p* < 0.05* versus* control, ** *p* < 0.01* versus* control, t-test.

For the wet starvation test, 30 day-old flies were provided with only water but no food, and their survival rates were measured after 20 h. While most of the males in the control group were still viable, we observed a statistical decrease in the survival rate of experimental males, which correlated with the increased theanine concentration (Figure 4A). However, no significant difference was noted among the female groups (Figure S2B).

To investigate the resistance against dry starvation, 30 day-old flies were deprived of both food and water, and their survival was recorded after 15 h. The survival rate of males decreased dramatically with increased theanine concentration (Figure 4B). Again, such an effect of theanine did not apply to females (Figure S2C).

It was shown that administration of green tea powder in the diet (2%–4%) significantly suppressed the lipid metabolism and fatty accumulation in mice, and thereby controlled the food intake and body weight [32]. Another study reported that oral intake of 130 mg green tea powder per day suppressed body weight increase without affecting food intake [37]. As a major component of green tea, theanine also could decrease the serum concentrations of triglycerides and non-esterified fatty acids, as well as the weight of intraperitoneal adipose tissues, thus significantly reducing the body weight in mice [31]. It was shown that theanine could induce dopamine release and reduce serotonin concentration in the brain [38,39], and both neurotransmitters are involved in the regulation of food intake [40]. For instance, amphetamine, a drug that increases brain dopamine concentration, has been used as an anti-obesity medicine in the U.S. because it can diminish the appetite and somehow produce anorexia. In this regard, theanine might change the neurotransmitters in the brain to exert its influence on body weight control [31,37]. In the present study, we measured the body weight of 30 day-old *Drosophila*, and noticed a slight, but not significant, decrease in males after theanine treatment (Figure S1G). However, no similar effect was observed in females (Figure S1H), likely due to the fact that females are generally much larger than males in size with a higher volume of water and fat content, and thus, might be less sensitive to the effects of theanine administration.

It is intriguing that theanine executed opposite effects in males, enhancing locomotor activity and courtship ability while reducing starvation tolerance. One plausible explanation is that theanine may improve the lipid metabolism and energy consumption in males, which promotes the mobility at the cost of energy storage. It has been known that energy expenditure and basal metabolic rate in males are higher than females among different age groups in mammals [41,42,43], which explains the phenomena that males are usually more active than females but less enduring under harsh conditions.

### 2.4. Theanine Diminishes the UV tolerance of Drosophila Females

**Figure 5:**
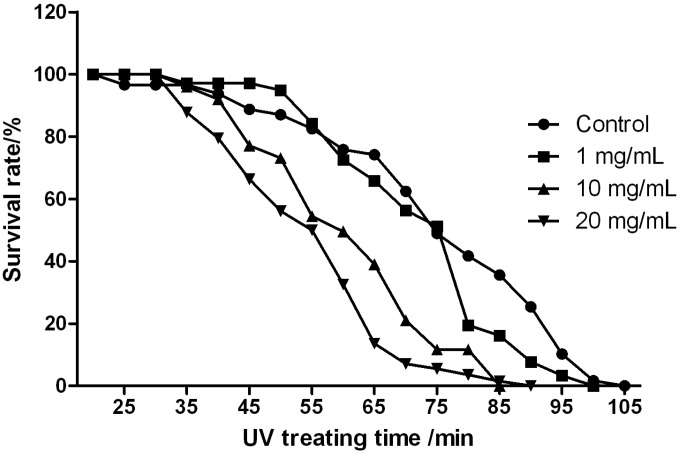
l-Theanine treatment decreases UV-tolerance of *Drosophila* females. 10 mg/mL and 20 mg/mL theanine significantly reduce the survival rates compare to control group when analyzed with long-rank test; ** *p* < 0.01* versus* control.

To examine the resistance against UV radiation, 30 day-old flies were exposed to 254 nm UV radiation in a UV cross-linking apparatus, and the survival rates were recorded every 5 min. We found that the tolerance of female flies against UV declined with the increase of theanine concentration (Figure 5), while the casualties in males were too high to allow us reach a conclusive result (data not shown).

### 2.5. Theanine Does not Affect the Developmental Circle of Drosophila

**Figure 6:**
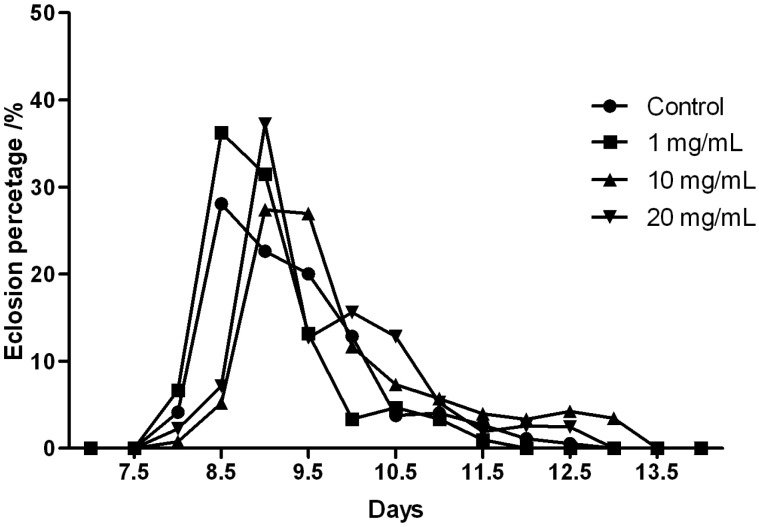
Effect of theanine on *Drosophila* development.

*Drosophila* development includes four stages: embryo, larva, pupa and adult. Under optimal conditions, it takes 9 to 10 days for a fertilized egg to complete the developmental cycle and emerge as an adult fly. To evaluate the safety profile of theanine on animal development, we allowed fertilized eggs to complete their developmental cycle in culture media with or without theanine, and recorded their eclosion time from pupa case. We found the eclosion peak was slightly postponed by the medium and high concentration of theanine, but not by the low concentration (Figure 6).

However, such effect was not statistically significant in Two-way ANOVA, suggesting theanine has no toxic effect on *Drosophila* development.

### 2.6. Theanine has no Effect on Drosophila Lifespan, Weight, Resistance against Heat and Anoxia

We also examined the effect of theanine on lifespan, weight, tolerance of heat and anoxia. We did not observe any distinct differences in male and female lifespan between each group (Figure S1A and B). Although males exhibited a slight decrease in the weight with increased theanine concentration (Figure S1G), the differences are not statistically significant, and no difference was observed in females (Figure S1H). Furthermore, theanine treatment did not produce any significant effect on males and females’ resistance against heat (Figure S1C and D) or anoxia (Figure S1E and F).

## 3. Experimental

### 3.1. Drosophila Strain

The Oregon-R strain, obtained from the Bloomington stock center (Bloomington, IN, USA), was used in all experiments. Flies were reared on a standard cornmeal agar medium at 25 °C on a 24-h light dark cycle.

### 3.2. l-Theanine Preparation and Administration

l-Theanine, synthesized by microbial fermentation and purified to 99%, was purchased from Southern Yangtze University Biotech Co., (Wuxi, China; http://www.wxjdbaitai.com/en_cha.asp).

l-Theanine solutions (0 mg/mL, 1 mg/mL, 10 mg/mL or 20 mg/mL) were mixed with dry yeast powder (solution:yeast = 5 mL:2 g, with acetic acid 10 μm/mL) and painted evenly onto the surface of the medium.

The flies were collected within 8 h after eclosion, males and females were segregated into different vials, with each vial containing about 20 flies. All flies were randomly selected into four groups, reared on medium with yeast paste containing no (0 mg/mL, served as control) or 1 mg/mL, 10 mg/mL or 20 mg/mL of l-theanine. Flies were transferred to fresh medium every three days.

### 3.3. Effect of l-Theanine on Drosophila Locomotion

On the fortieth day, the flies were transferred into a 25 cm long glass tube and allowed 5 min for adaptation. Their climbing height within 20 s was recorded and the median was taken as the result. Those who remained at the bottom were marked as 0 cm; those who fell after crawling to a certain height were recorded by the maximum height they had reached.

### 3.4. Effect of l-Theanine on Drosophila Male Courtship

On the thirtieth day, each male was paired with five 3-day-old virgin females that have been reared on standard medium, and the courtship behavior within 2 h was recorded.

### 3.5. Effect of l-Theanine on Drosophila Wet Starvation Tolerance

On the thirtieth day, the flies were transferred into an empty vial containing a piece of filter paper soaked with water, and their mortality was recorded after 20 h.

### 3.6. Effect of l-Theanine on Drosophila Dry Starvation

On the thirtieth day, the flies were transferred into an empty vial, and their mortality was recorded after 15 h.

### 3.7. Effect of l-Theanine on Drosophila UV Tolerance

On the thirtieth day, the flies were placed in Ø5 cm Petri dishes covered by plastic film, and subsequently placed into the UV cross-linking apparatus (wavelength = 254 nm). The flies were allowed to suspended two min after every five min of exposure, and the survival rate was recorded. The data is converted into a percentage and shown as an average survival curve.

### 3.8. Effect of l-Theanine on Drosophila Eclosion Time

Five pairs of 3-day-old flies were placed in a vial, allowed to mate and lay eggs on medium for 6 h at 25 °C. Each group includes ten vials, and eclosed flies were counted every 6 h. The number of eclosed flies in every 12 h was converted into a percentage.

### 3.9. Effect of l-Theanine on Drosophila Lifespan

The number of dead flies was counted every three days till all flies died. The data was converted into a percentage and shown in a survival curve.

### 3.10. Effect of l-Theanine on Drosophila Weight

On the thirtieth day, flies were collected in 1.5 mL centrifuge tubes and the weight was measured. The average weight was calculated as (total mass—mass of empty tube)/number of flies.

### 3.11. Effect of l-Theanine on Drosophila Anoxia Tolerance

**Figure 7:**
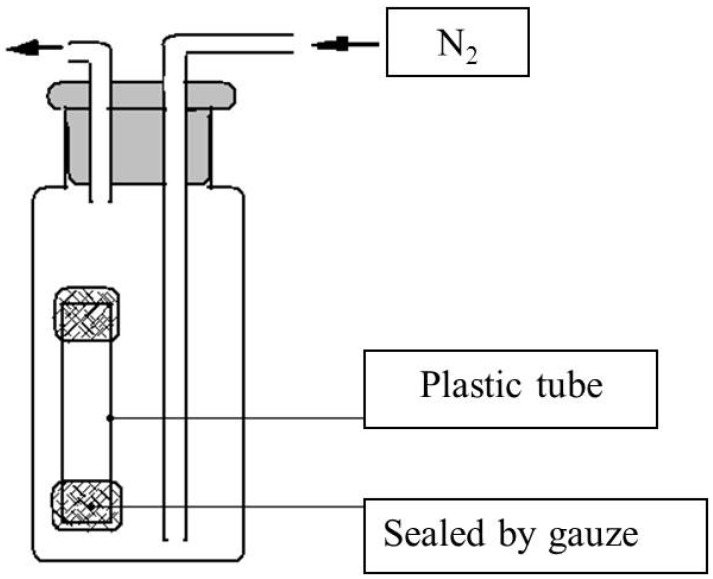
Schematic diagram of apparatus to test anoxia tolerance.

The device was set up as in Figure 7. Thirty day-old flies were collected in an Ø 10 mm × 10 mm transparent plastic tube and subsequently placed into the bottle. The bottle was filled with 0.3 L/min of pure nitrogen to maintain the oxygen level at less than 0.03%, and timing started after 5 min. Males were released from the bottle after 2 h, and females after 2.5 h. The treated flies were recovered on normal medium for 72 h and their survival rate was recorded.

### 3.12. Effect of l-Theanine on Drosophila Heat Tolerance

On the thirtieth day, flies were periodically placed at 37 °C for fifteen min and recovered at 25 °C for ten min till all of them died. The number of dead flies was counted while recovering, and the data was converted into a percentage and shown in an average survival curve.

### 3.13. Statistical Analysis

Student’s t-test was used for statistical evaluation in locomotion, starvation tolerance, weight, anoxia test. Results are expressed as the mean ± SE, and the differences were considered significant at *p* < 0.05 and extremely significant at *p* < 0.01.

The log-rank test was carried out to evaluate different survival rates between control and experiment groups subjected to UV or heat challenge, or in the lifespan assays. The differences were considered significant at *p* < 0.05 and extremely significant at *p* < 0.01.

Chi square test was applied to evaluate the trend of male courtship behavior. The differences were considered significant at *p* < 0.05 and extremely significant at *p* < 0.01. Two-way ANOVA was used for statistical analysis of *Drosophila* development cycle.

## 4. Conclusions

In this report, we examined the physiological effects of l-theanine, an amino acid from green tea, on *Drosophila melanogaster*. We found that theanine was able to produce a number of effects on animal behavior and resistance against stress conditions. In particular, theanine was able to boost male locomotor and courtship ability while diminishing their starvation tolerance. In females, theanine could impair their resistance against UV radiation. It is intriguing that theanine could elicit diverse responses, presumably through different organs, in a sex-dependent manner. The observed effects of theanine on *Drosophila* need to be confirmed in other animal models, and should be investigated further to reveal the underlying mechanism(s). 

## Supplementary Materials

Supplementary materials can be accessed at: http://www.mdpi.com/1420-3049/18/11/13175/s1.
