# Supplementary material for: Physiological Effects of l-Theanine on Drosophila melanogaster
Source: Molecules. 2013 Oct 24;18(11):13175–87. doi: 10.3390/molecules181113175 (PMC6270322; doi:10.3390/molecules181113175)

Supplementary Materials

Figure S1. Effect of theanine on lifespan of males (**A**) and females (**B**); heat tolerance of males (**C**) and females (**D**); anoxia resistance of males (**E**) and females (**F**); and body weight of males (**G**) and females (**H**).

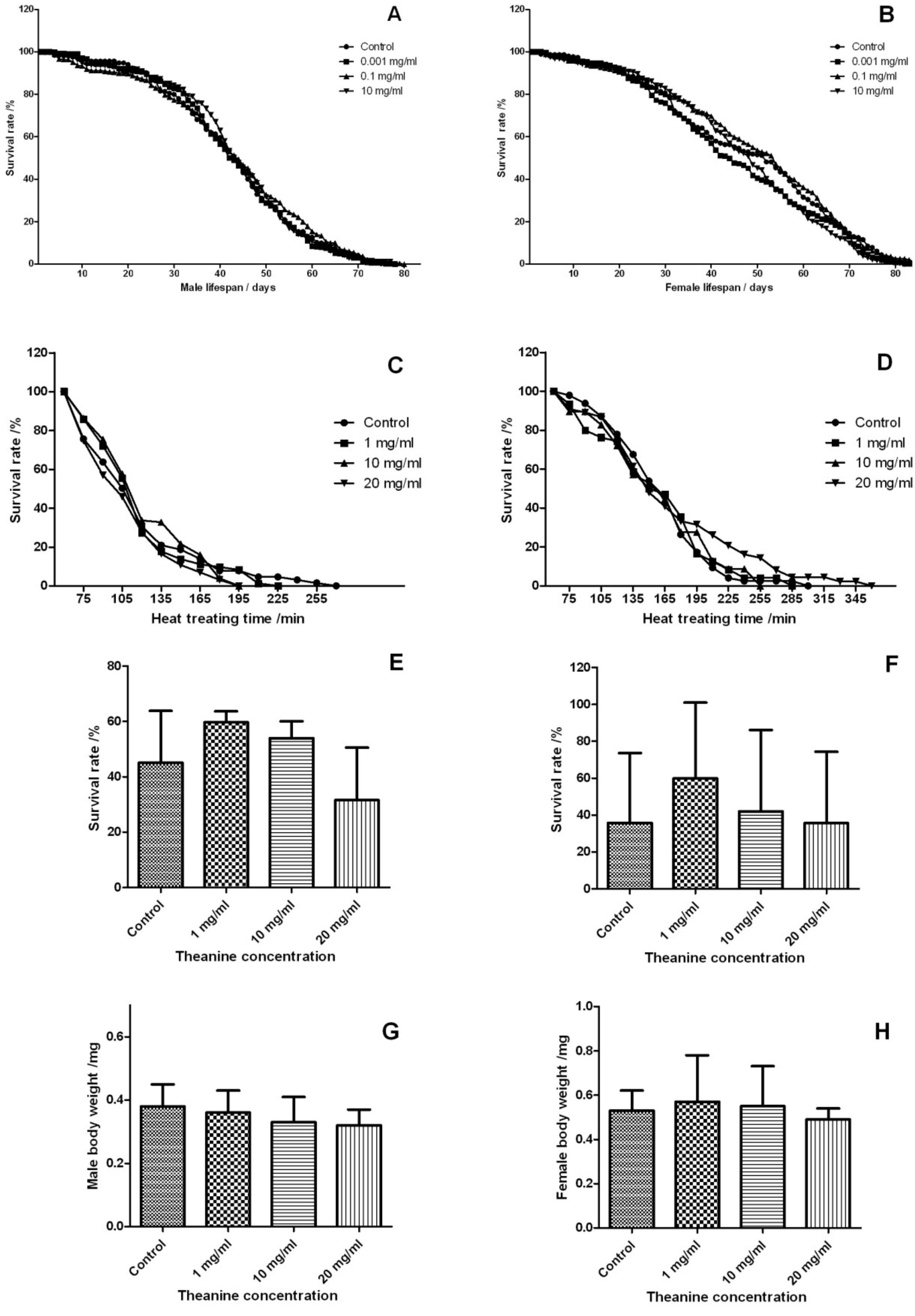

Figure S2. Theanine has no significant effect on climbing ability (**A**), resistance against wet (**B**) and dry (**C**) starvation in females. $p > 0.05$ *versus* control, t-test.

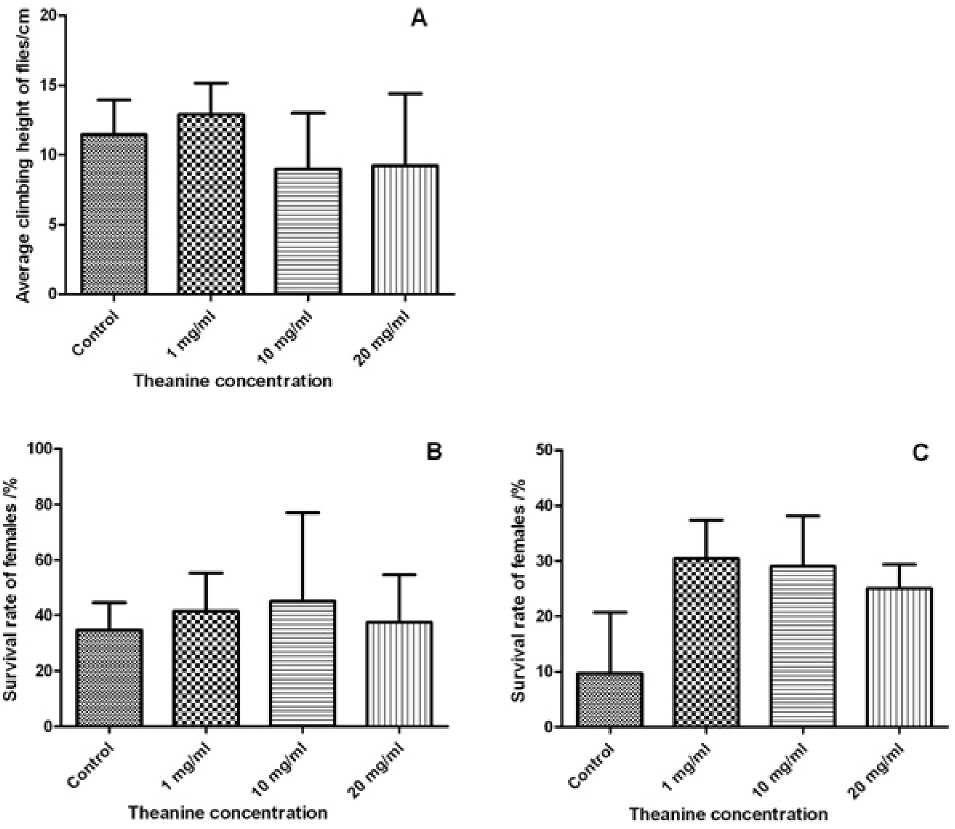

Supplement: Supplementary file 1 [file molecules-18-13175-s001.pdf]
